# The role of probiotics, prebiotics and synbiotics in animal nutrition

**DOI:** 10.1186/s13099-018-0250-0

**Published:** 2018-06-06

**Authors:** Paulina Markowiak, Katarzyna Śliżewska

**Affiliations:** Department of Biotechnology and Food Sciences, Institute of Fermentation Technology and Microbiology, Lodz University of Technology, ul. Wólczańska 171/173, 90-924 Lodz, Poland

**Keywords:** Animal health, Prebiotics, Probiotic bacteria, Synbiotics

## Abstract

Along with the intensive development of methods of livestock breeding, breeders’ expectations are growing concerning feed additives that would guarantee such results as accelerating growth rate, protection of health from pathogenic infections and improvement of other production parameters such as: absorption of feed and quality of meat, milk, eggs. The main reason for their application would be a strive to achieve some beneficial effects comparable to those of antibiotic-based growth stimulators, banned on 01 January 2006. High hopes are being associated with the use of probiotics, prebiotics and synbiotics. Used mainly for maintenance of the equilibrium of the intestinal microbiota of livestock, they turn out to be an effective method in fight against pathogens posing a threat for both animals and consumers. This paper discusses definitions of probiotics, prebiotics and synbiotics. Criteria that have to be met by those kinds of formulas are also presented. The paper offers a list of the most commonly used probiotics and prebiotics and some examples of their combinations in synbiotic formulas used in animal feeding. Examples of available study results on the effect of probiotics, prebiotics and synbiotics on animal health are also summarised.

## Background

It is estimated that by 2050 the number of people in the world will reach 9 billion. Constant growth of the human population is inseparably associated with a growing demand for food of plant and animal origin. For that reason, scientists are looking for solutions allowing intensification of food production, with simultaneous reduction of production costs, and in compliance with high standards of quality and safety (for both people and the environment). Types of used feed additives affect animal health and increased production of high quality meat, eggs, milk and fish. Animal production is inseparable from nutrition and health of the consumer, and animal intestinal pathogens, such as *Campylobacter*, *Salmonella*, *Listeria* and *Yersinia*, are a direct source of food contamination and a cause of zoonoses. Therefore, new methods of animal breeding are introduced, aimed at increased quality and safety of meat, while taking animal welfare and respect for the natural environment into account.

Both animal feed and feed supplements have to meet some strict criteria, without a simultaneous rise of animal breeding costs. In the past, antibiotics and other medicinal products had been broadly used, mainly in order to modify the alimentary microbiota and to boost productivity and animal growth. Long-term use of those substances has led to development of drug-resistant microorganisms, posing a threat to consumers’ health and exerting a negative effect on the environment [1, 2]. As a result, on 1 January 2006 the use of antibiotic-based growth stimulators was banned in the European Union. Therefore, alternative natural substances ensuring similar effects have been sought. The Regulation (EC) No. 1831/2003 of the European Parliament and of the Council of 22 September 2003 on additives used in animal nutrition, mentions probiotics and prebiotics among other substances, whereas in the Regulation (EC) No. 767/2009 on the placing on the market and use of feed, this aspect was not changed. High hopes are also evoked in relation to the synergistic combination of both these components, namely the so-called synbiotics.

## Probiotics

The term “probiotic” comes from two Greek words (“pro” and “bios”) and it means “for life”. The first concept of probiotics was probably suggested in 1907 by Mechnikov [3], who noted that bacteria may have a beneficial influence on the natural intestinal microflora. The term of “probiotic” was probably invented by Ferdinand Vergin, who in his paper of 1954 entitled “Anti- und Probiotika” compared a harmful effect of antibiotics and other antimicrobial agents on the intestinal microbiota with a beneficial effect (“probiotica”) of selected bacteria [4]. With time, the definition of probiotic was largely modified (Table 1).

**Table 1:** Definitions of probiotics

Year	Definitions
1965	A substance secreted by one microorganism which stimulates the growth of another [5]
1971	Tissue extracts which stimulate microbial growth [6]
1974	Organisms and substances that contribute to intestinal microbial balance [7]
1989	Live microbial feed supplement which beneficially affects the host animal by improving microbial balance [8]
1992	Viable mono- or mixed culture of live microorganisms which, applied to animals or man, have a beneficial effect on the host by improving the properties of the indigenous microflora [9]
1996	A live microbial culture or cultured dairy product that beneficially influences the health and nutrition of the host [10]
1996	Living microorganisms which, upon ingestion in certain numbers, exert health benefits beyond inherent basic nutrition [11]
1998	Living microorganisms that on ingestion in certain numbers exert health benefits beyond inherent basic nutrition [12]
1999	A microbial dietary adjuvant that beneficially affects the host physiology by modulating mucosal and systemic immunity, as well as improving nutritional and microbial balance in the intestinal tract [13]
2001	A preparation of or a product containing viable, defined microorganisms in sufficient numbers, which alter the microflora (by implantation or colonization) in a compartment of the host and by that exert beneficial health effect in this host [14]
2002	Live strains of strictly selected microorganisms which, when administered in adequate amounts, confer a health benefit on the host [15]
2004	Preparation of viable microorganisms that is consumed by humans or other animals with the aim of inducing beneficial effects by qualitatively or quantitatively influencing their gut microflora and/or modifying their immune status [16, 17]
2009	Live microorganisms, which when administered in adequate amounts, confer a health benefit on the host [18]
2013	Live strains of strictly selected microorganisms which, when administered in adequate amounts, confer a health benefit on the host [19]

The current definition formulated in 2002 by FAO and WHO working group experts states that probiotics are “live strains of strictly selected microorganisms which, when administered in adequate amounts, confer a health benefit on the host” [15]. The definition was in 2013 maintained by the International Scientific Association for Probiotics and Prebiotics (ISAPP). The term “probiotic’ is reserved for formulas or products that meet some strictly defined criteria. The most important of these criteria include: an appropriate count of viable cells, a beneficial effect on a host’s health (which may also involve stimulation of growth), and a beneficial effect on the function of the alimentary tract. Efficacy of probiotic preparations depends on numerous factors. For that reason proper selection of bacterial strains and application of a correct dose are highly important. Due to their beneficial effect on health and stimulation of growth, probiotics are broadly used in animal feeds, particularly for pigs and poultry. That type of formulas may contain one or more selected strains of microorganisms, and depending on the species and age of host animals they may be administered as a powder, suspension, capsules, pellet, gel or paste. They are used periodically or constantly, directly per os or as an additive to feed and premixes. Probiotic cultures used as feed additives must meet some specific criteria.

### Selection criteria and requirements for probiotic strains

Assessment of the safety of probiotic strains is necessary for the optimization of their use. However, it is not an easy task [20]. The mode of action of probiotics as microbial additives to feed is not fully understood. By adhering to the alimentary tract probiotic organisms may survive difficult conditions, and offer a beneficial effect on the stability and protection of the intestinal ecosystem. They also influence the course of digestive and metabolic processes and the immunological response. Consequently, properties of probiotics lead to improved health of animals, increased productivity [21], and improved immunity of the host [22].

The immunomodulatory mechanism action of probiotics involved in animal health and diseases is particularly important and is based on innate or adaptive immune system. The gastro-intestinal lumen contains essential nutrients and beneficial microorganisms, but also pathogen microorganisms, toxins, and some foreign antigens [23, 24]. Epithelial cells in the GIT mucosa create a selectively permeable barrier between the lumen environment and the internal body tissues [25]. This barrier is the first line of host defense against harmful microbes in the GIT (gut innate immunity) but factors such as stress or disease conditions can disrupt this barrier [24, 26, 27]. Certain probiotic microorganisms can enhance the function of intestinal barrier through modulation of the phosphorylation of cytoskeletal and tight junction proteins and thereby influencing the intestinal mucosal cell–cell interactions and also cellular “stability” [24, 28]. Restitution of the GIT mucosa barrier function by probiotics has been observed in both in vitro and in vivo models [29, 30]. The mechanism can be related to the alterations in the secretion of mucus or chlorides, or the changes in the expression of tight junction proteins by epithelial cells, however the details of this mode of action are still not very clear [28, 31]. In other side, animals can adaptive immune system. Animal immune responses should be stimulated in some cases (for example, in infection and immune-deficiency situations) but be suppressed in others (for example, in allergy and autoimmune disease situations) [32]. Research has showed that the normal gut microbiota by stimulating gastrointestinal immune response (antibody production and increasing phagocytic activity) can support animal’s defense systems against invading pathogens [33]. Fuller [34] explained two ways in which the immune system is stimulated: they can either migrate through the gut wall as viable cells or multiply to a limited extent, and the antigens released by the dead organisms are absorbed and directly stimulate the host immune system. It is the product of this change that further induces the immune response [33].

**Fig. 1:**
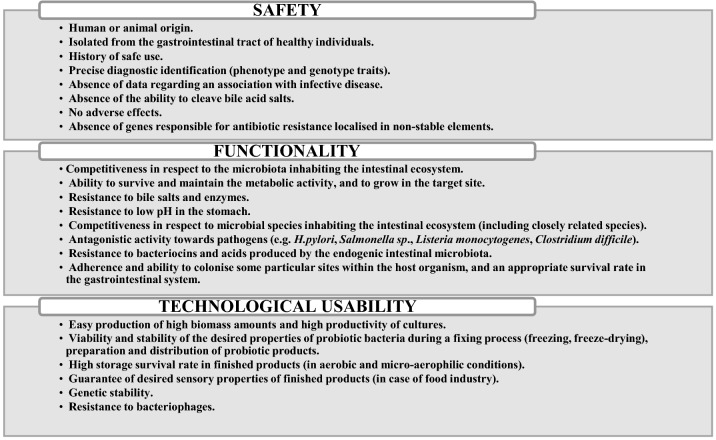
Selection criteria and required properties of probiotic strains [15, 37]

Selection of new probiotic organisms involves strains and even geni of microorganisms demonstrating the most beneficial or the most specific effects. The assessment focuses mostly on safety and the benefit-to-risk ratio associated with the use of a given probiotic strain. Microorganisms used for production of probiotic animal formulas should be isolated from individuals belonging to the species for which they are intended, because part of health beneficial effects is probably species specific. Due to that procedure, the obtained biological material is maximally adapted to the conditions present in the alimentary tract of the given species of animals [35]. Moreover, probiotic cultures added to feed should be resistant to temperatures and pressures used in the process of pelleting, and to humidity and the effect of adverse substances during feed handling and storage, such as heavy metals or mycotoxins. The period of high activity of probiotics in feed and premixes must not be shorter than 4 months [35]. To extend that period, formulas are encapsulated, which results in extended survival of strains [36]. According to the suggestions of the WHO, FAO and the European Food Safety Authority (EFSA), in their selection process probiotic strains must meet both safety and functionality criteria, and those related to their technological usefulness (Fig. 1).

### Probiotic microorganisms

Probiotic products may contain one or more selected microbial strains. Microorganisms used as feed supplements in the EU are mostly bacteria. Most often they are Gram-positive bacteria belonging to the following geni: *Bacillus*, *Enterococcus*, *Lactobacillus*, *Pediococcus*, *Streptococcus*. Also some fungi and yeast strains of *Saccharomyces cerevisiae* and *Kluyveromyces* species are probiotics. Bacteria belonging to the geni *Lactobacillus* and *Enterococcus* are components of the natural microbiota of the animal alimentary tract, and are usually present in amounts of 10^7^–10^8^ and 10^5^–10^6^ CFU/g, respectively. On the other hand, yeast and *Bacillus* genus bacteria are not usually present in the gastrointestinal system. Majority of the abovementioned microorganisms should be potentially safe for the host. However, some of them may pose problems; e.g. *Enterococcus* genus bacteria may participate in transmission of antibiotic resistance, and *Bacillus cereus* strain are able to produce endotoxins and emetic toxins [38].

In 2005, only 13 of 21 probiotic products were approved as feed supplements in the EU to be used in piglets, and some in sows and porkers [39]. As many as seven of those products contained *Enterococcus faecium* strains (a natural environment of the gastrointestinal tract), two of them contained *Bacillus* genus spores (most commonly occurring in soil), another two contained *Saccharomyces cerevisiae* yeast strains, and only one contained *Lactobacillus farciminis* and *Pediococcus acidilactici* strains occurring in the gastrointestinal tract and in dairy products, respectively [39]. Therefore, the origin of strains that may potentially be used as feed supplements may be various. It is important, however, that probiotic organisms are present in appropriate amounts. The recommended dose for the majority of probiotic strains is 10^9^ CFU/kg of feed [39].

Probiotics are subject to regulations contained in the general food law, and according to them, they should be safe for human and animal health. In the USA, microorganisms used for consumption purposes should have the Generally Regarded As Safe (GRAS) status, regulated by the FDA. In Europe, EFSA introduced the term of Qualified Presumption of Safety (QPS). The QPS concept involves some additional criteria of the safety assessment of bacterial supplements, including the history of safe usage and absence of the risk of acquired resistance to antibiotics [38, 40]. Importantly, GRAS status is applied to microorganisms and microbial-derived ingredients used in food products while QPS is applied to any biological agent in the form of bacteria, fungi, or virus, that is intentionally added at different stages into the food chain. Probiotic use may help decrease the rate of development of antibiotic-resistant strains secondary to widespread and rampant antibiotic use [41, 42]. On the other hand, some microorganisms used as probiotics are not exempted from acquiring antibiotic resistance genes. In views of their shared microbial environment in the gastrointestinal tract of animals, a risk of pathogenic microbes acquiring antibiotic resistance genes from probiotic microbes exists, and vice versa. If improperly cooked, livestock fed of probiotics that are consumed by humans as food may also pose as a possible source of antibiotic resistance genes for the human gut microbiota [43]. Therefore, given the emerging risk of spreading antibiotic resistance genes through probiotic strains, the QPS is considered by many as the more applicable and flexible probiotics criteria [44]. Table 2 presents probiotic microorganisms contained in animal feed supplements.

**Table 2:** Probiotic microorganisms mostly intended for animals [45–47]

Type *Lactobacillus*	Type *Bifidobacterium*	Other lactic acid bacteria	Other microorganisms
*L. brevis* ^a^	*B. animalis* ^a^	*Enterococcus faecalis*	*Bacillus cereus*
*L. casei* ^a^	*B. longum* ^a^	*Enterococcus faecium*	*Bacillus licheniformis* ^a^
*L. crispatus* ^a^	*B. pseudolongum*	*Lactococcus lactis* ^a^	*Bacillus subtilis* ^a^
*L. farciminis* ^a^	*B. thermophilum*	*Leuconostoc citreum* ^a^	*Propionibact. Freudenreichi* ^a^
*L. fermentum* ^a^		*Leuconostoc lactis* ^a^	*Saccharomyces cerevisiae (boulardi*)^a^
*L. murinus*		*Leuconostoc mesenteroides* ^a^	*Saccharomyces pastorianus* ^a^
*L. gallinarium* ^a^		*Pediococcus acidilactici* ^a^	*Kluyveromyces fragilis*
*L. paracasei* ^a^		*Pediococcus pentosaceus* ^a^	*Kluyveromyces marxianus* ^a^
*L. pentosus* ^a^		*Streptococcus infantarius*	*Aspergillus orizae*
*L. plantarum* ^a^		*Streptococcus salivarius*	*Aspergillus niger*
*L. reuteri* ^a^		*Streptococcus thermophilus* ^a^	
*L. rhamnosus* ^a^		*Sporolactobacillus inulinus*	
*L. salivarius* ^a^			

^a^ QPS microorganisms

Ready-made probiotic formulas for animals usually contain one, two, or more strains of microorganisms [48]. Effectiveness of that type of formulas is affected by numerous factors, including: proper selection of strains, and unitary dose containing an appropriate count of viable cells. To preserve the properties of probiotic formulas, they have to be stored and used as recommended by their manufacturers. Due to the content of viable microorganisms, probiotic formulas are susceptible to unfavourable conditions, such as temperature and light. It is important that no other substances are used while probiotics are administered, and that water used for dilution contains no chlorine or other disinfectants. Water with the formula should be administered to animals within 6–12 h. An interval of 24–48 h between the end of antibiotic therapy or administration of any other antimicrobial agents and the onset of the probiotic therapy is also important. Formulas containing many ingredients (the highest number of microbial species) are usually the most effective [49]. Examples of probiotic formulas available in the market are presented in Table 3.

**Table 3:** Examples of probiotic formulas used in nutrition of livestock [50–52]

Trade name of the preparation (producer)	Microorganisms	Destination
Acid-Pak-4-Way (Alltech)	*Lactobacillus acidophilus, Enterococcus faecium*	Poultry, pigs
Anta Pro EF (Dr. Eckel)	*Enterococcus faecium*	Pigs
Avian PAC (Soluble Loveland Industries)	*Streptococcus faecium, Lactobacillus acidophilus,*	Poultry
Biogen D (Bio-Gen)	*Bifidobacterium bifidum, Lactobacillus acidophilus, Pediococcus faecium*	Poultry
Biogen N (Bio-Gen)	*Bifidobacterium bifidum, Lactobacillus acidophilus, Pediococcus faecium*	Pigs
Biogen T (Bio-Gen)	*Bifidobacterium bifidum, Lactobacillus acidophilus, Enterococcus faecium*	Pigs
Bio Plus2B^®^ (Chr. Hansen)	*Bacillus subtilis, Bacillus licheniformis*	Pigs, calves, poultry
BioPlus^®^YC (Evonik Industries)	*Bacillus licheniformis, Bacillus subtilis*	Pigs
B.I.O.Sol (Biochem)	*Enterococcus faecium*	Poultry
Bro-biofair (Vitality Co.)	*Saccharomyces servisia*	Pigs
Calsporin (ORFFA)	*Bacillus subtilis*	Poultry, pigs
Cerbiopor	*Lactobacillus: acidophilus, brevis, casei, fermentum, lactis, plantarum; Bacillus: subtilis, megaterium, pumilus; Enterococcus faecium, Cellulomonas sp., Saccharomyces cerevisiae*	Pigs
Cernivet LBC (Cerbios)	*Enterococcus faecium*	Calves, pigs
Cerbiogalli	*Lactobacillus: acidophilus, casei, plantarum*	Poultry
Cylactin (DSM)	*Enterococcus faecium*	Poultry, pigs, calves
Doctor Em^®^ (Biotron)	*Lactobacillus: paracasei, plantarum*; *Lactococcus lactis*, *Saccharomyces cerevisiae*	Poultry, pigs, calves
Ecobiol (Norel Animal Nutrition)	*Bacillus amyloliquefaciens*	Poultry
Enviva™ Pro (DANISCO Animal Nutrition)	*Bacillus subtilis*	Poultry
Enviva^®^MPI (DANISCO Animal Nutrition)	*Lactobacillus: farciminis, rhamnosus*	Pigs
Farmaflore soluble (Farm’apro)	*Lactobacillus: rhamnosus*, *farciminis*	Poultry
FloraMax-B11 (Pacific Vet Group)	*Lactobacillus salivarius, Pediococcus parvulus*	Poultry
GalliPro^®^ (Evonik Industries)	*Bacillus subtilis*	Poultry
Galvit Probiotyk (Galvit)	*Enterococcus faecium*	Poultry
Lactiferm	*Enterococcus faecium*	Pigs, poultry, calves
Lavipan^®^ (JHJ)	*Lactobacillus: plantarum, casei; Lactococcus lactis, Carnobacterium divergens, Saccharomyces cerevisiae*	Poultry, pigs
LSP 122 (Alpharma)	*Bacillus licheniformis*	Pigs
Microguard (PeterLab Holdings)	*Bacillus: licheniformis, megaterium, mesentricus, polymyxa, subtilis; Saccharomyces boulardii; Bididobacterium bifidum; Lactobacillus: acidophilus, bulgaricus, plantarum; Streptococcus faecium*	Poultry, pigs
MicroSource S (Agtech Products Inc.)	*Bacillus: subtilis, licheniformis*	Pigs
Oralin^®^ (Chevita GmbH)	*Enterococcus faecium*	Pigs, calves, poultry
PrimaLac (Star Labs, Inc.)	*Bifidobacterium: bifidium, thermophilus; Enterococcus faecium; Lactobacillus: acidophilus, casei,*	Pigs, beef, dairy, horses, poultry, deer
Probiomix	*Bifidobacterium bifidum Lactobacillus amylovorus Enterococcus faecium*	Calves, poultry
Probion (Woogene B&G Co. Ltd.)	*Bacillus subtilis, Clostridium butyricum, Lactobacillus acidophilus*	Pigs, poultry
Probios (Chr Hansen)	*Lactobacillus: acidophilus, casei, plantarum, lactis; Enterococcus faecium; Bacillus subtilis*	Poultry, pigs
Probiosacc C-I	*Saccharomyces cerevisiae*	Calves
Pro-Biotyk em15^®^ (ProBiotics)	*Bacillus subtilis, Bifidobacterium: animalis, bifidum, longum, Lactobacillus: acidophilus, casei, delbrueckii subsp. bulgaricus, fermentum,.plantarum; Lactococcus lactis subsp. lactis; Saccharomyces cerevisiae; Streptococcus thermophilus*	Poultry, pigs, calves, horses
Propoul (International Company s.r.o.)	*Lactobacillus fermentum*	Poultry
Protexin (Protexin Probiotics International Ltd.)	*Lactobacillus: plantarum, delbruecki* subsp*. bulgaricus, acidophilus, rhamnosus; Bifidobacterium bifidum; Streptococcus salivarius* subsp*. thermophilus; Enterococcus faecium; Aspergilus oryzae; Candida pintolepesii*	Poultry, pigs, sheep, cattle,
Provita LE (Schaumann)	*Lactobacillus rhamnosus, Enterococcus faecium*	Pigs, calves
Super-CyC (Choong Ang Biotech Co. Ltd.)	*Bacillus subtilis, Saccharomyces cerevisiae*	Poultry, cattle, horses, pigs
Toyocerin^®^ (Rubinum S.A.)	*Bacillus toyonensis*	Pigs
UltraCruz (Santa Cruz Animal Health)	*Enteroccus faecium, Lactobacillus: acidophilus, casei, plantarum*	Cattle, calves, poultry
Yea Sacc (Alltech)	*Lactobacillus rhamnosus, Enterococcus faecium*	Cattle, calves

### Probiotics in animal breeding

Farm animals are exposed to the environment-related stress (e.g. rearing methods, diet, etc.). Various factors may cause disturbance of balance in the intestinal ecosystem and may become risk factors for pathogenic infections. Regardless of the species, animal health is crucial for the production chain. The use of probiotics in animal feeding is associated with their verified efficacy in modulation of the intestinal microbiota. Administration of probiotic strains, both individual and combined, may have a significant effect on absorption and utilisation of feed, daily increase of body weight and total body weight of various animals, including turkeys [53], chicken [54], piglets [55, 56], sheep, goats [57], cattle, and horses [58]. An addition of probiotic microorganisms to feed results in improved quantity and quality of milk, meat and eggs [59]. Moreover, probiotics reduce the effect of weak limbs in broiler chicken [60]. In the case of piglets, the main expected effect of probiotics is a reduction of frequency of diarrhoea, posing a problem in initial post-weaning weeks. The efficacy of probiotics in combating diarrhoea is one of the most commonly studied aspect. Recombined probiotics are one of the most novel biomedical applications of genetically modified organisms (GMO) [59]. The absence of clinical side effects is an important benefit of probiotics.

In the case of pig production, weaning is the critical moment, when animals are the most exposed to stress (nutrition changes from milk to the diet based on vegetable polysaccharides). Also the environment is changed, as a result of transfer to a production farm. All those factors may disturb immunological functions and have a negative effect on the balance of pigs’ intestinal microbiota [61]. Böhmer et al. [62] used a feed with an addition of a supplement of the *Enterococcus faecium* DSM 7134 probiotic strain in feeding of 33 sows between the 90th day of pregnancy and the 28th day of lactation. A significant improvement of feed consumption, offspring size and weight of studied animals was observed. Improved feed consumption and productivity may be helpful in prevention of the so called “starvation sterility” of young sows, caused by reduced feed consumption along with mobilisation of body tissue and insufficient energy during lactation [62]. Probiotics have a positive effect on various digestions processes, especially on the cellulolytic ones, and the synthesis of microbial proteins [63]. Mountzouris et al. [64] studied the efficacy of a probiotic formula containing two strains of *Lactobacillus* genus, and one strain of each geni: *Bifidobacterium*, *Enterococcus*, *Pediococcus*, compared to a product containing avilamycin. The experiment was conducted on 400 broiler chickens, for 6 weeks. It was found that administration of the probiotic caused stimulation of animal growth comparable to the effect of treatment with the avilamycin-containing product. Moreover, the addition of the formula to feed and/or water for chickens caused a significant probiotic effect by modulation of the composition and activity of the intestinal microbiota [64].

A favourable effect of feed supplemented with the YEA-SACC-1026 probiotic [65] and with bacterial strains *Bacillus licheniformis* and *Bacillus subtilis* [65] on the quality of milk (fat and protein content) and increased body weight of lambs was also confirmed. The probiotic was used during the late period of pregnancy and during milk feeding. Other studies indicated that the addition of the Bio Plus 2B^®^ probiotic containing *Bacillus subtilis* and *Bacillus licheniformis* strains caused a significant improvement of sows’ blood parameters (higher cholesterol and total lipid levels) and milk parameters (higher content of milk fat and protein) during milk-feeding [66]. Yu et al. [67] determined the effect of steamed corn with the addition of *Aspergillus oryzae* culture in cows’ diet on their milk productivity. The experiment was carried out on 32 cows for 70 days. It was confirmed that the addition of *A.oryzae* to steamed corn resulted in an increased percentage of protein and dry fat-free solids (Solids-Not-Fat, SNF) in milk. Studies completed by Ceslovas et al. [68] focused on the effect of probiotics: YEASTURE, MICROBOND and of phytobiotics: YUCCA, QUILLAYA on the growth of pigs and quality of meat. It was found that the studied probiotics contributed to increased carcase production in the group of experimental animals. Moreover, those formulas had also an effect on improved culinary properties of pork, reduced loss on cooking and improved tenderness of meat. However, no significant improvement of daily body weight increase and carcase production was found in groups fed with phytobiotics compared to the control.

Moreover, probiotics contribute to increased production and improved quality of eggs [69, 70], and to reduced *Salmonella* contamination in eggs [71]. In the studies completed by Haddadin et al. [69] chickens were fed with a feed with a supplement of *Lactobacillus acidophilus* for 48 weeks. Based on obtained results, it was concluded that egg production and feed conversion levels were significantly higher in experimental animals compared to the control group of animals. A reduced cholesterol level was also noted in egg yolks from animals fed with the probiotic strain. The researchers suggested that the latter effect was a reflection of lower serum cholesterol levels in studied birds. Kurtoglu et al. [70] determined the effect of the commercial formula Bio Plus^®^2B on daily feed consumption, egg productivity and weight, specific gravity, feed conversion ratio, serum and egg yolk cholesterol and chicken serum triglycerides. The experiment was carried out on 480 chickens, using various doses of probiotic (depending on the study group) for 90 days. It was found that probiotic supplementation at the doses of 250, 500 and 750 mg/kg of feed caused increased production of eggs, and reduced egg damage ratio. Serum and egg yolk cholesterol levels also became reduced in probiotic-fed animals. Moreover, in the case of probiotic doses of 500 and 750 mg/kg of feed, a reduced triglyceride level was found in the serum of experimental animals, compared to the control group. On the other hand, the probiotic used in doses of 250 and 500 mg/kg of feed had a positive impact on the feed conversion ratio [70].

Studies also confirmed a favourable effect of probiotics on improved growth of farm animals, including cows [72], young calves, piglets [73] and broiler chickens [74]. Kyriakis et al. [73] demonstrated efficacy of the LSP 122 probiotic containing spores of *Bacillus licheniformis* in combating diarrhoea syndrome occurring in piglets in 3–10 days post weaning (post-weaning diarrhoea syndrome, PWDS) in relation to clinical symptoms, mortality, body weight gain and feed conversion. The principal cause of morbidity and mortality of newborn piglets and recently weaned pigs is infection with enterotoxic strains of *Escherichia coli* (ETEC). A lower frequency and intensity of diarrhoea was observed in animals receiving feed with an addition of a probiotic. Moreover, mortality of all pigs receiving supplementation with probiotics was significantly lower compared to the negative control (fed with non-modified feed).

A positive effect of probiotics compared to the negative control was determined based on data regarding the assessment of body weight increase and the feed conversion ratio. The summary of all results obtained in the study by Kyriakis et al. [73] indicated that the LSP 122 probiotic used at the dose of 107 viable spores of *Bacillus licheniformis* is useful in combating PWDS caused by ETEC.

An addition of probiotic microorganisms to animal feed plays a significant role in the fight against pathogens, including: *Listeria monocytogenes*, *Salmonella* Typhimurium, and in protection of piglets against diarrhoea [75]. In the case of chickens, the role of probiotics was demonstrated in protection against the following pathogens: *Escherichia coli* [76], *Salmonella* [77], *Campylobacter* [77], *Clostridium* and *Eimeria* [78]. Chateau et al. [76] studied the antagonistic properties of *Lactobacillus* ssp. strains isolated from commercial probiotic products, in relation to bacterial strains pathogenic for chickens (including the serotypes of *Listeria monocytogenes*, *Escherichia coli* and *Salmonella*). Growth inhibition of all pathogens was observed as a consequence of presence of one or a combination of several studied probiotic bacteria. The most pronounced inhibition was observed in relation to *Listeria monocytogenes*, but a satisfactory inhibition was also observed for *Escherichia coli*, *Salmonella* Typhimurium and *Salmonella* Enteritidis. Stern et al. [77] compared the efficacy of the CE culture used for elimination of *Salmonella* spp. infections (competitive exclusion) and of the MCE culture (mucosal competitive exclusion) used for combating of *Campylobacter* colonisations in broiler chickens. 210 chicks were studied. The results indicated that the microbiota of 90 birds treated with the CE culture was much more colonised by *Salmonella* Typhimurium than in 90 chicks treated with the MCE culture. Also in the case of colonisation with the *Campylobacter* genus bacteria, a superior effect of the MCE culture was found compared to the animals treated with the CE culture.

In summary, probiotics increase the control of pathogenic microorganisms in poultry, thanks to which they can prevent diseases such as salmonellosis, campylobacteriosis or coccidiosis [52, 79, 80]. In addition, diarrhea infections caused by enterotoxic *E. coli* strains is one of the major health problems in pigs in the post-weaning period. As a result, they cause significant economic losses by increasing mortality, decreasing the growth rate and related veterinary costs [81]. There is a positive effect of probiotics not only on reducing the frequency of diarrheas, but also on the alleviation of their course. Such effects are described, among others after the use of preparations containing *Bacillus licheniformis* [73] or *B. toyonensis* [82, 83]. Probiotic bacteria such as *Lactobacillus sobrius* [84] or *Lactobacillus paracasei* [85] have been shown to limit intestinal colonization by pathogenic *E. coli.*

There are reports indicating that the use of bacterial probiotics is more effective in the case of chickens, pigs and young calves, whereas administration of probiotic yeast strains (*Saccharomyces cerevisiae*) and fungi (*Aspergillus oryzae*) offers better results in adult ruminants [86].

*Salmonella* Enteritidis bacteria colonise the gastrointestinal tract of poultry and cause food-related diseases in humans. Reduced colonisation of *Salmonella* Enteritidis in the poultry alimentary tract causes reduction of the potential contamination of carcases, thus offering an improved quality of consumed meat. Tellez et al. [74] studied the effect of specific probiotics combined with specific antibodies against *Salmonella* Enteritidis, *Salmonella* Typhimurium and *Salmonella* Heidelberg on the colonisation of intestines and invasion of organs by *Salmonella* Enteritidis in broiler chickens, and also on the body weight of studied animals [73]. The results of the study indicate that the combination of probiotic strains: *Lactobacillus acidophilus*, *Streptococcus faecium* with bacterial strains *Salmonella* Enteritidis, *Salmonella* Typhimurium and specific antibodies against *Salmonella* Heidelberg exerts a favourable effect on reduced *Salmonella* Enteritidis colonisation in the bodies of broiler chickens at the productive age.

According to Simon [39], approximately 80% of experiments performed in order to combat diarrhoea in piglets, regardless of the applied probiotic microorganism (*Bacillus cereus*, *Enterococcus faecium*, *Pediococcus acidilactici*), confirmed a positive effect of those probiotics. Based on the experiment lasting for 6 weeks on three groups of piglets (two fed with a feed with an addition of a probiotic containing the *Enterococcus faecium* NCIMB 10415 genus bacteria and one with an addition of *Bacillus cereus toyoi*) the author concluded that modification of microbiota resulting from the activity of the probiotic *Enterococcus faecium* NCIMB 10415 bacteria caused a significant reduction in the frequency of diarrhoea, compared to the control group, with an overall positive effect on the health of sows and piglets. The author did not observe any significant effect on animal growth. The probiotic had also effect on the function of epithelial tissues and on immunological response (a significantly reduced level of cytotoxic T cells (CD8+) in piglets’ jejunal epithelium). Based on those observations, the author concluded that the applied bacterial strain may potentially replace antibiotic-based stimulants used in sow and piglet breeding [39].

When summing up the advantages of probiotic use, one should emphasise the role of probiotics in protection of animals against pathogens, enhancement of immunological response, reduced need for antibiotic-based growth stimulants, and high safety of those formulas. An increasing demand for meat products is currently observed, and consumers’ expectations are reflected in producers’ strive for the highest possible quality of meat. The use of feed supplementation with non-chemical formulas, such as probiotics, may meet that expectation. Table 4 lists the examples of results of studies on the effects of probiotic microorganisms in animal nutrition.

**Table 4:** Examples of trials regarding the effect of probiotics on animal health

Reference	Subjects	Microorganism	Time of administration	Main outcome
Absorption and utilisation of feed, diarrhoea, body weight gain				
[87]	114 Piglets	*E. faecium* DSM 10,663 NCIMB 10415	From birth to weaning (24 ± 3.2 days)	Reduced portion of subjects suffering from diarrhoea, improving performance as indicated by a higher daily weight gain
[53]	118 Turkeys	Probiotic FM-B11 (*Lactobacillus*)	For 3 days post birth and after approx. 6 weeks of life	Use of the selected commercial probiotics resulted in increased market BW and reduced cost of production
[54]	308 Broiler chickens	*E. faecium* NCIMB 10415	21 days	Confirmed efficacy of supplementation in relation to chicken body weight gain and FCR
[57]	20 Growing maltese goat kids	*L.acidophilus*, *L. salivarius*, *L.reuteri*	7 months	Improved metabolic activity, body weight and proportions in animals receiving a probiotic
[64]	400 Broiler chickens	*Lactobacillus* (2 strains), *Bifidobacterium*, *Enterococcus, Pediococcus*	6 weeks	Stimulated growth, comparable to the avilamycin-containing product (ASW)
[62]	33 Sows	*E. faecium* DSM 7134	From the 90th day of pregnancy to the 28th day of lactation	A significant improvement of feed consumption, offspring size and weight of studied animals
Intestinal ecosystem imbalance, pathogenic infections				
[88]	153 Healthy piglets and 26 sows	*E. faecium* NCIMB 10415	17 weeks (sows), 6 weeks (piglets)	Reduced pathogenic bacterial (*E. coli*) load of healthy piglets and sows
[89]	6 Piglets	*L. plantarum* Lq80	21 days	Increased total gut populations of lactobacilli in weaned pigs
[56]	15 Pigs	2 strains of *L. murinus*, and one of each: *L. salivarius* subsp. *salivarius*, *L. pentosus*, *P. pentosaceous*	30 days	Animals treated with probiotics showed reduced incidence, severity, and duration of diarrhoea. The administered probiotic bacteria improved both the clinical and microbiological outcome of *Salmonella* infections
[77]	210 Chickens	CE culture MCE culture	No data	Significantly lower colonisation of the intestinal microflora of experimental animals fed with CE by *Salmonella* Typhimurium and *Campylobacter*, compared to the group of animals fed with MCE
[39]	Sows and piglets	*E. faecium* NCIMB 10415, *B. cereus toyoi*	6 weeks	Modification of microflora as a result of the action of the *E. faecium* strain caused a significant reduction of frequency of diarrhoea in comparison to the control group. The probiotic had also effect on the function of epithelial tissues and on immunological response (significantly reduced level of cytotoxic T cells (CD8+) in piglets’ jejunal epithelium)
Improved quality of meat, milk, eggs				
[90]	Lambs	Probiotic YEA-SACC-1026	During pregnancy and milk-feeding	A positive effect on the quality of milk (fat and protein content) and increased body weight of lambs
[65]	Lambs	*B. licheniformis, B. subtilis*	During pregnancy and milk-feeding	A positive effect on the quality of milk (fat and protein content) and increased body weight of lambs
[66]	109 Sows during milk-feeding	Probiotic Bio Plus 2B (*B. licheniformis, B. subtilis)*	From the day of allocation (14 days prior to the expected farrowing) up to the weaning day	A significant improvement of blood parameters (higher cholesterol and total lipid level) and of milk parameters (higher fat and protein content) during milk feeding in sows
[67]	32 Cows	*A. oryzae*	70 days	The effect on the increased ratio of protein and SNF in milk
[70]	480 Chickens	Probiotic Bio Plus 2B (*B. licheniformis*, *B. subtilis*)	90 days	Increased production of eggs and reduced ratio of damaged eggs in probiotic-fed animals. At appropriate doses: reduced level of serum and egg-yolk cholesterol. Reduced serum triglyceride levels compared to the control and a positive effect on FCR

*ASW* antibiotic-based growth stimulator, *CE* competitive exclusion, *FCR* feed conversion ratio, *MCE* mucosal competitive exclusion

## Prebiotics

Besides probiotics, also prebiotics are used as natural feed additives. Already in 1921 Rettger and Cheplin reported that after consumption of carbohydrates the human intestinal microbiota was enriched with lactic bacteria [91]. The prebiotic concept was first initiated in 1995 [92]. The concept has evolved since (Table 5). The currently used definition was created in December 2016 by the International Scientific Association of Probiotics and Prebiotics (ISAPP). The definition says that the group of prebiotics may involve other substances besides carbohydrates (such as polyphenols and polyunsaturated fatty acids transformed into corresponding conjugated fatty acids), and may act not only in the alimentary tract. Another important aspect is that they are no longer limited to human food, but may be also considered in other categories, such as animal nutrition. On the other hand, requirements concerning selective mechanisms of modulation of microbiota as well as the condition of documented beneficial effects on the health of the host have been maintained [93].

**Table 5:** Definitions of prebiotics

Year	Definitions
1995	“Non-digested food components that, through stimulation of growth and/or activity of a single type or a limited amount of microorganisms residing in the gastrointestinal tract, improve the health condition of a host” [92]
2004	“A selectively fermented component allowing specific changes in the composition and/or activity of microorganisms in the gastrointestinal tract, beneficial for host’s health and wellbeing” [96]
2007	“A nonviable food component that confers a health benefit on the host associated with modulation of the microbiota” [97]
2010	‘Dietary prebiotics’ as “a selectively fermented ingredient that results in specific changes in the composition and/or activity of the gastrointestinal microbiota, thus conferring benefit(s) upon host health” [98]
2015	“A non-digestible compound that, through its metabolization by microorganisms in the gut, modulates the composition and/or activity of the gut microbiota, thus, conferring a beneficial physiological effect on the host” [99]
2016	“A substrate that is selectively utilized by host microorganisms conferring a health benefit” [93]

Many different nutrients, such as pectins, cellulose and xylanes, favour development of various intestinal microorganisms. Prebiotics should not be extensively metabolised, but should induce targeted metabolic processes, thus bringing health benefits to the host’s ecosystem. The best documented benefits are associated with the use of indigestible oligosaccharides, such as fructans and galactans [94]. That phenomenon is explained by, among others, easy degradability of bonds present in the structure of fructo-oligosaccharides (FOS) and galacto-oligosaccharides (GOS) by certain enzymes, such as β-fructanosidase and β-galactosidase, commonly occurring in *Bifidobacterium* genus bacteria. Some types of nutritional fibre may be considered prebiotic. Prebiotics play a significant role in nutrition of both livestock and home pets. When assessing the effect of prebiotics on health, one has to take into account the fact that all groups of animals mentioned above differ in terms of anatomy, physiology, nutrition, intestinal microbiota and habitat [95].

### Prebiotic selection criteria

In order to determine and demonstrate that a substance is a potential prebiotic, one has to indicate its source, origin, purity, chemical composition and structure. Prebiotics has to cover safe regulations required by all nations, such as posses Generally Recognized As Safe (GRAS) status, proper dose and side effects evaluation, no contaminants and impurities, do not alter intestinal microbiota to obtain negative effects on the host. It is emphasized that the term prebiotic may be used only when beneficial health effect related to the microbiota modulation in a specific site [97].

**Fig. 2:**
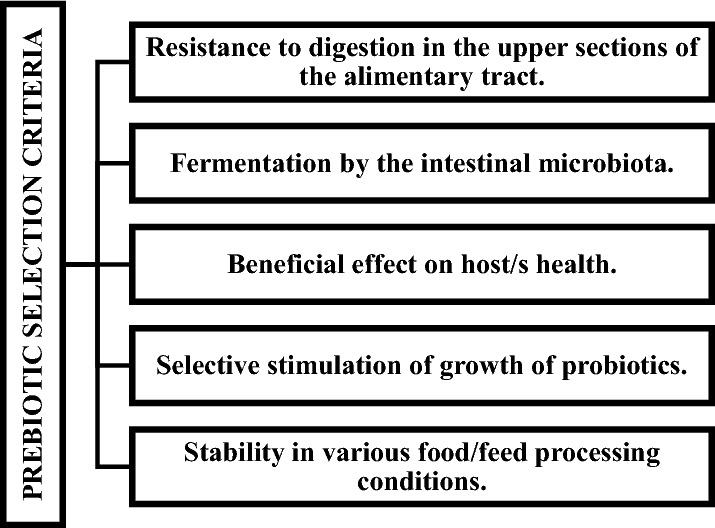
Requirements for potential prebiotics [100, 101]

According to Wang [100], there are five basic criteria for classification of food components as prebiotics (Fig. 2). First of all, it is assumed that prebiotic substances must be resistant to digestion in the upper sections of the alimentary tract. As a result, prebiotics reach the large intestine, where they become selectively fermented by potentially beneficial intestinal bacteria (the second criterion). The fermentation may lead to changes in metabolic processes, and to improved operation of the immunological system, thus exerting a beneficial effect on the host’s health (the third criterion). Very important is selective stimulation of growth of the probiotic bacteria (another criterion). Also technological features of prebiotics, associated with their successful manufacturing and availability for bacterial metabolism in the intestine, are important (the last criterion).

### Prebiotic substances

Among prebiotic substances there are: non-absorbable carbohydrates (oligosaccharides and polysaccharides), peptides, proteins, and lipids. Legumes, fruit and cereals are natural sources of prebiotics. However, many similar substances are synthesized using industrial chemical and enzymatic methods [101]. Some commonly used prebiotics are: FOS, oligofructose, trans-galacto-oligosaccharides (TOS), gluco-oligosaccharides, glico-oligosccharides, lactulose, lactitol, malto-oligosaccharides, xylo-oligosaccharides, stachyose and raffinose [102–106]. When they reach the large intestine, those substances become nutritional substrates for beneficial intestinal bacteria [107]. In terms of properties that determine a favourable effect on the host’s health, prebiotics may be divided into following groups: not digested (or only partially digested), not absorbed in the small intestine, poorly fermented by bacteria in the oral cavity, well fermented by seemingly beneficial intestinal bacteria and poorly fermented by potential pathogens in the bowel [108]. Prebiotics most commonly used in livestock nutrition are [108, 109]: FOS, GOS, inulin, isomalto-oligosaccharides (IMO), xylo-oligosaccharides (XOS), lacticol, lactulose, cereal fibre. When designing the composition of prebiotic formulas, determination of an appropriate dosage is essential. Overdose of prebiotics may lead to flatulence and diarrhoea. On the other hand, a great advantage of that kind of formulas is that they may be used for a long time and preventively, having no adverse effects noted for antibiotics. Table 6 presents examples of formulas used in livestock nutrition and containing prebiotic substances.

**Table 6:** Examples of prebiotic formulas available in the market and intended for livestock

Trade name of preparation (producer)	Prebiotic substances	Destination
Bacto CS1000	Polysaccharides, oligosaccharides	Poultry
BionatStart	MOS, β-glucans	Calves
DOLSORB DN (Dolfos)	MOS, β-glucans	Poultry
MetSac MOS (VITTRA)	MOS, β-glucans	Calves, pigs, poultry
Mycocyd forte (Herbiline)	β-glucans	Poultry
Mycostop (Extra-vit)	MOS, β-glucans	Poultry, pigs
PROFEED^®^ (Beghin Meiji)	scFOS	Horses, pigs, poultry, calves

*FOS* fructo-oligosacharides, *MOS* malto-oligosacharides, *scFOS* short chain fructo-oligosaccharides

### Prebiotics for animals

Various feed additives are used in studies on the effect of prebiotics on the gastrointestinal microbiota and general health condition of pigs. Smiricky-Tjardes et al. [110] administered TOS at the dose of 35 g/kg feed to pigs for 6 weeks. A significant increase of stool *Bifidobacterium* and *Lactobacillus* count was found compared to the control group. Tzortzis et al. [111] used a novel blend of GOS produced as a result of activity of galactosyl transferase in *Bifidobacterium bifidum* 41171 bacteria. The administration of that prebiotic to pigs at the dose of 40 g/kg feed in a 3-step system of continuous culture caused a significant increase of *Bifidobacterium* count and of acetic acid level, with simultaneous reduction of intestinal pH, compared to the control group and the diet with an addition of inulin. Moreover, the studied blend of oligosaccharides caused a strong inhibition of adhesion of *Escherichia coli* (ETEC) and *Salmonella enterica* serotype Typhimurium to HT29 cells in in vitro studies [111]. An interesting study was also carried out on the effect of barley and oat varieties with different composition of carbohydrates on the intestinal microbiota of 72 weaned piglets, for 15 days. It was found, that the increased β-glucan levels and changes in the ratio of amylopectin and amylose led to a selective modulation of growth of butyric acid bacteria which is able to hydrolyse some complex carbohydrates, such as xylan or β-glucan. Therefore, differences between cereal varieties in form and amount of carbohydrates had an effect on piglets’ intestinal microbiota, and an appropriate selection of cereals had a positive effect on *Bifidobacterium* and *Lactobacillus* count.

Xu et al. [124] checked effects of FOS used in doses: 0, 2, 4 and 8 g/kg feed on the activity of digestive enzymes and on intestinal morphology and microbiota. It was found that the administration of FOS at the dose of 4 g/kg feed had a positive effect on the mean daily growth of studied animals, and on the growth of *Bifidobacterium* and *Lactobacillus* bacteria, with a simultaneous inhibition of growth of *Escherichia coli* in chickens’ gastrointestinal tract. On the other hand, in the study by Juśkiewicz et al. [112] carried on turkeys for 8 weeks, no effect of FOS used at concentrations of 0.5, 1 and 2% was found on animal growth and productivity. However, reduction of the intestinal pH was noted in case of FOS administration at the concentration of 2%. Supplementation of broiler chickens’ diet with prebiotics results in reduction of gastrointestinal pH and increased *Lactobacillus* and *Bifidobacterium* counts, caused by increased amount of volatile fatty acids [113]. In their study, Yusrizal and Chen [114] checked the effect of feeding broiler chickens with fructane (of chicory origin) containing feed on growth of birds and length and structure of the intestine of studied animals. The experiment was conducted on 96 broiler chickens, for 6 weeks. An improved body weight gain, feed turnover and reduced serum cholesterol were found. Moreover, feed supplementation with fructanes caused increase of *Lactobacillus* genus bacteria count and reduction of counts of potential pathogens, such as *Salmonella* and *Campylobacter* in the broiler chicken gastrointestinal tract [114]. In their study, Kleessen et al. [115] bred 380 chickens for 35 days, giving them drinking water with an addition of artichoke-based fructane-containing (0.5%) syrup. The effect of fructane supplementation on the animals’ intestinal microbiota was studied. It was observed that the addition of fructanes to drinking water caused a reduction of *Clostridium perfringens* count, and a decrease in the level of bacterial endotoxin. Stanczuk et al. [116] analysed the effect of addition of inulin and MOS administered to turkeys ad libitum in two different concentrations (0.1 and 0.4%) as a feed supplement, during the period of 8 weeks of rearing. No increased feed consumption or higher body weight of turkeys were observed. However, in prebiotic-fed groups a higher concentration of SCFA was observed compared to the control group. In other studies conducted by Sims et al. [117] on 180 turkeys bred for 18 weeks, a supplementation of feed with MOS resulted in better growth of study animals. Spring et al. [118] studied the effect of administration of *Saccharomyces cerevisiae* yeast containing MOS in their cellular wall on reduction of count of various intestinal pathogens in chickens. It was observed that the administration of MOS-containing yeast resulted in a reduced count of *Salmonella* in chicks’ intestines by 26%, compared to control animal receiving a non-modified diet. Studies completed by Thitaram et al. [119] verified the effect of isomalto-oligosaccharides (IMO) administered in the following concentrations: 1, 2 and 4% (by weight) on intestinal microbiota of broiler chickens infected with *Salmonella* Typhimurium. Supplementation of animal feed with IMO caused a significant reduction of *Salmonella* Typhimurium count. While chewing, digestion and effectiveness of the administered feed were not significantly different from the control group. It was also observed that the addition of IMO to feed caused an increase in *Bifidobacterium* genus bacteria count. Moreover a significant loss of weight was observed in the case of birds fed with 1% IMO compared to control animals fed with the non-modified feed [119]. In other studies, Biggs et al. [121] focused on the effect of feeding chicks with feed with addition of 5 different oligosaccharides (inulin, oligofructose, MOS, short-chain oligosaccharide and TOS) [120]. No significant increase in body weight was observed in any case. Moreover, the study demonstrated that excessively high prebiotic dose may have a negative impact on the gastrointestinal system and delay the process of growth of animals [120]. Similarly, other studies completed by Jung et al. [122] on broiler chickens demonstrated that administration of feed with an addition of GOS at various concentrations for 40 days of rearing had no effect on the feed conversion index, body weight and consumption of feed [121]. Nevertheless, the addition of the prebiotic had a positive effect on the increase of *Bifidobacterium* bacteria in intestines of study chickens. Summing up, the main effect of prebiotics on health of chickens consists in an increased count of *Bifidobacterium* and reduced intestinal colonisation by pathogenic bacteria [122, 123]. Results of studies on the effect of prebiotics on animal health are often contradictory, which is a result of high specificity of individual compounds, various doses and time of application. Table 7 presents the examples of studies on the effect of prebiotics on animal health.

**Table 7:** Examples of trials regarding the effect of prebiotics on animal health

Reference	Subjects	Prebiotic	Time	Main outcome
Absorption and utilisation of feed, diarrhoea, body weight gain				
[124]	240 Broiler chickens	FOS	49 days	Administration of fructooligosaccharides at the dose of 4 g/kg feed had a positive effect on the mean daily growth of studied animals, and on growth of *Bifidobacterium* and *Lactobacillus* bacteria, with a simultaneous inhibition of growth of *Escherichia coli* in experimental animals’’ gastrointestinal tract
[112]	320 Turkeys	FOS	8 weeks	No effect on growth and productivity of experimental animals. However, reduction of the intestinal pH was noted in case of FOS administration at the concentration of 2%
[125]	96 Broiler chickens	Fructans from chicory	6 weeks	An improved body weight gain, feed turnover and reduced serum cholesterol
[116]	40 Turkeys	MOS, inulin	8 weeks	No increased feed consumption or higher body weight of experimental animals were observed. A higher SCFA concentration was found in animals fed with prebiotics, compared to the control
[117]	180 Turkeys	MOS	18 weeks	Improved growth of experimental animals
[120]	120 Chickens	Inulin, oligofructose, MOS, short-chain oligosaccharide, TOS	21 days	No significant body weight gain. The study demonstrated that an excessively high prebiotic dose may have a negative impact on the gastrointestinal system and delay the process of growth of animals
Intestinal ecosystem imbalance, pathogenic infections				
[110]	12 Pigs	TOS	6 weeks	A significant increase of stool *Bifidobacterium* and *Lactobacillus* count compared to the control group
[111]	40 Weaned male pigs	GOS	Mean of 34 days	A significant increase of *Bifidobacterium* genus bacteria count and of concentration of acetic acid, with simultaneous reduction of intestinal pH compared to the control group, and the diet with an addition of inulin. Moreover, the GOS supplementation caused a strong inhibition of adhesion of *Escherichia coli* (ETEC) and *Salmonella enterica* serotype Typhimurium to HT29 cells in in vitro studies
[114]	98 Broiler chickens	Fructans from chicory	6 weeks	The supplementation with fructans caused an increase *Lactobacillus* genus bacteria count and reduction of count of potential pathogens, such as *Salmonella* and *Campylobacter*
[115]	380 Chickens	Fructans from artichoke	35 days	Reduced *Clostridium perfringens* count and bacterial endotoxin level
[119]	120 Broiler chickens infected with *Salmonella* Typhmiurium	IMO	21 days	A significant reduction of *Salmonella* Typhimurium count. Chewing, digestion and effectiveness of the administered feed were not significantly different from the control. group. A significant loss of weight in case of animals fed with 1% IMO compared to the control group. The supplementation with IMO caused an increase of the *Bifidobacterium* count in the gastrointestinal system of experimental animals
Improved quality of meat, milk, eggs				
[126]	350,560 Eggs from Ross 308 broiler	DiNovo (DN; laminarin and fucoidan), Bi2tos (BI; non-digestive TOS)	42 days	No significant differences in the final count of chickens, mortality, breeding density (kg/m^3^), FCR, European Broiler Index between all experimental groups. The administration of DN and BI resulted in a minor increase (P > 0.05) of the mean BW and a minor improvement (P > 0.05) of FCR in the BI group. Chickens exposed to DN and BI demonstrated a significant increase of BW, carcase weight, weight of the myocardium and weight of the breast, compared to the control group. Summing up, the administration of prebiotics in ovo resulted in an improvement of many parameters significant for the commercial production of poultry

*BW* body weight, *FCR* feed conversion ratio, *FOS* fructo-oligosaccharides, *GOS* galacto-oligosaccharides, *IMO* isomalto-oligosaccharides, *MOS* manno-oligosaccharides, *TOS* transgalacto-oligosaccharides

## Synbiotics

Also formulas containing both probiotics and prebiotics are used in animal nutrition. In 1995, Gibson and Roberfroid introduced the term of “synbiotic” by specifying in this way “a mixture of probiotics and prebiotics that beneficially affects the host by improving the survival and implantation of live microbial dietary supplements in the GI tract, by selectively stimulating the growth and/or activating the metabolism of one or a limited number of health-promoting bacteria, and thus improving host welfare” [92]. As the word “synbiotic” implies synergy, the term should be reserved for those products in which a prebiotic component selectively favours a probiotic microorganism [127]. The principal purpose of that type of combination is improvement of survival of probiotic microorganisms in the gastrointestinal tract. Synbiotics have both probiotic and prebiotic properties and were created in order to overcome some possible difficulties in survival of probiotics in the gastrointestinal tract [128]. Probiotics beneficially influence the intestinal equilibrium, and constitute a protective barrier for the alimentary tract. Prebiotics, on the other hand, supply energy and nutrients for probiotic bacteria [129, 130]. Therefore, an appropriate combination of both components in a single product should ensure a superior effect, compared to the activity of the probiotic or prebiotic alone [131, 132]. The health effect of synbiotics is probably associated with the individual combination of a probiotic and prebiotic [133]. Considering a huge number of possible combinations, the application of synbiotics for modulation of intestinal microbiota in animals seems promising [134].

### Synbiotic selection criteria

Most of all, probiotic strains and prebiotics considered in the process of designing a synbiotic formula should meet all the criteria presented in “Selection criteria and requirements for probiotic strains” and “Prebiotic selection criteria”. When composing the synbiotic formula, selection of probiotics and prebiotics that have a beneficial effect on the host’s health when used separately is crucial. When selecting probiotic substances, it is helpful to determine their potentially beneficial properties for the metabolism of a probiotic. A formula may be considered a synbiotic if a selective stimulation of growth of beneficial microorganisms is confirmed, along with no or limited stimulation of growth of other microbes. Also technological aspects should be considered. Determination of composition of a synbiotic formula is an extremely difficult task, requiring many studies.

### Synbiotics in use

Previous sections discussed probiotic microorganisms and prebiotic substances most commonly used in animal nutrition. A combination of *Bifidobacterium* or *Lactobacillus* genus bacteria with FOS in synbiotic products seems to be the most popular. Table 8 presents examples of synbiotic formulas available in the market, and used for livestock nutrition.

**Table 8:** Examples of commercial synbiotic formulas used in nutrition of livestock

Trade name of the preparation (producer)	Microorganisms	Prebiotic substances	Destination
Biomin^®^IMBO (ME BIOMIN GmbH)	*Enterococcus faecium*	FOS	Poultry, pigs, calves
DigestAid™	*Pediococcus acidilactici, Saccharomyces: cerevisiae, boulardii*	β-glucan, MOS	Horses
PoultryStar^®^ (ME BIOMIN GmbH)	*Bifidobacterium animalis, Enterococcus faecium, Lactobacillus: reuteri, salivarius, Pediococcus acidilactici,*	Inulin	Poultry
Synbiotic poultry (Vetafarm)	*Lactobacillus: acidophilus, casei, salivarius, plantarum, rhamnosus, brevis, Bifidobacterium: bifidum, lactis, Streptococcus thermophilus*	Inulin	Poultry

*FOS* fructo-oligosacharides, *MOS* mannano-oligosacharides

### Synbiotics for animals

The animal gastrointestinal tract, besides being the environment for a huge number of microorganisms, plays also a significant immunological role and constitutes the most important barrier protecting the host from toxins, pathogens, and consequences of their action, namely inflammation. Currently available data regarding effects of synbiotic on animal health are insufficient and require further studies. However, they clearly indicate the effective synergistic action of probiotics and prebiotics in reduction of populations of bacterial gastrointestinal pathogens.

Recent years have seen a remarkable evolution in the development and applications of traditional and DNA-based molecular tools that are allowing the microbiologists to characterize and understand the microbial communities in unprecedented ways [135]. Metagenomic investigations, comprising isolation of entire microbial community genomes, construction and screening of clone libraries, enable the microbiologists to have a look at a more complete scenario of an environmental microbial communities, and thus, to better understand the microbes–environment interactions [136]. Metagenomics could be a promising strategy for appraising of the synbiotics effect of the intestinal microbiota of animals.

Nemcová et al. [137] confirmed the synergistic effect of *Lactobacillus paracasei* bacteria combined with FOS in the intestinal microbiota of piglets. The researchers observed an increase of total anaerobic and aerobic count, and increased number of beneficial *Lactobacillus* and *Bifidobacterium* genus bacteria in the group of animals fed with a synbiotic. At the same time, the *Escherichia coli*, *Enterobacteriaceae* and *Clostridium* genus bacteria count decreased in the stool of studied piglets [137].

Lee et al. [113] in a 16 day experiment studied the effect of synbiotics on growth, digestibility of nutrients, emission of harmful gases and composition of intestinal microbiota of 150 pigs during the weaning period. Supplementation with the synbiotic product containing a combination of a probiotic originating from anaerobic microbiota (bacteria—10^9^ CFU/ml, yeast—10^5^ CFU/ml, moulds—10^3^ CFU/ml) and a prebiotic (MOS, sodium acetate, ammonia citrate) results in improved digestion of nutrients, reduced emission of harmful gases and prevents bacterial infections during the weaning period [138].

Mohnl et al. [139] observed that the synbiotic product (Biomin^®^ PoultryStar) had a comparable growth stimulating potential to avilamycin (an antibiotic-based growth stimulant) in broiler chicken. Vicente et al. [140] verified the effect of a synbiotic product containing *Lactobacillus* spp. with the addition of lactose. 320 turkeys infected with *Salmonella* were bred, and a positive effect of the synbiotic on feed conversion and body weight gain of study animals was demonstrated. Li et al. [141] assessed the effect of administration of FOS and *Bacillus subtilis* bacteria to broiler chickens. 720 broiler chickens were bred and improvement of the average daily growth and of the feed conversion ratio, as well as reduced incidence of diarrhoea and mortality of animals in comparison to animals treated with aureomycin (tetracycline antibiotic) were observed. During the administration of a combination of GOS and *Bifidobacterium lactis* bacteria to broiler chickens during a 40 day rearing period, a significant increase of *Bifidobacterium* and *Lactobacillus* count and in overall population of anaerobic bacteria was observed in the intestinal microbiota of the study animals. However, no effect on feed consumption and conversion, and on body weight was observed. Awad et al. [142] studied the effect of the synbiotic product containing *Enterococcus faecium* bacteria and FOS as a prebiotic, and immunomodulating substances from marine algae (ficophytic substances) on health of broiler chickens. 600 broiler chickens bred for 5 weeks were studied. A significant increase of the average daily body weight gain, carcase ratio and the feed conversion ratio was found in comparison to control animals. However, no effect of the synbiotic on body weight gain was observed, except for the small intestine, in which a significant growth of intestinal villi was observed within the duodenum and the ileum. Based on the study on 240 broiler chickens, it was found that probiotics and prebiotics have a favourable effect on performance parameters, during some terms even superior to antibiotics used for the comparison. Moreover, it was observed that prebiotic supplementation may be helpful in reduction of abdominal fat following 42 days of breeding. It was observed that probiotics and prebiotics may be possibly used as substitutes of antibiotic-based growth stimulants [143].

Summing up, researchers agree that synbiotic products provide a better efficacy compared to the separate application of probiotics and prebiotics [121, 142, 144, 145]. Table 9 lists the examples of results of studies focusing on the effect of synbiotics on animal health.

**Table 9:** Examples of trials regarding the effect of synbiotics on animal health

Reference	Subjects	Composition of synbiotic	Time	Main outcome
Absorption and utilisation of feed, diarrhoea, body weight gain				
[140]	320 Turkeys infected with *Salmonella*	*Lactobacillus* spp., lactose	14 days (trial 1–3), 18 days (trial 4)	The effect of a synbiotic on improved feed conversion and increased body weight of experimental animals
[141]	720 Broiler chickens	*B. subtilis*, FOS	6 weeks	Improved average daily growth, FCR, reduced incidence of diarrhoea and mortality, compared to animals treated with aureomycin
[142]	600 Broiler chickens	*E. faecium*, FOS	5 weeks	A significant increase of the average daily body weight gain, carcase ratio and FCR compared to the control
Intestinal ecosystem imbalance, pathogenic infections				
[146]	33 Conventional healthy sucking piglets	*L. plantarum*, maltodextrin and/or FOS	7 days	Reduced counts of *E. coli* O8:K88 in the jejunum and colon of piglets, and it was associated with increased acetate concentrations in the ileum and colon
[138]	150 Pigs during weaning	A probiotic of anaerobic microflora (bacteria/yeast/moulds), MOS, sodium acetate, ammonia citrate	16 days	Improved digestion of nutrients, reduced emission of harmful gases and prevention of bacterial infections during the weaning period
Improved quality of meat, milk, eggs				
[147]	58 Holsten dairy cows	*L. casei*, dextran	1 year	Significant increase in Holstein cow milk production; including total milk, fat, protein and solids-non-fat production

*FCR* feed conversion ratio, *FOS* fructo-oligosaccharides, *MOS* mannano-oligosaccharides

## Conclusions

Despite numerous difficulties associated with the registration of feed additives, particularly in the category of zootechnical feed additives, modern global economy and strong market competition result in the need to introduce new technologies to animal nutrition. Numerous scientific reports confirm a beneficial effect of probiotics on animal health, particularly in terms of protection against pathogens, stimulation of immunological response and increased production capacity. Prebiotics may be used alternatively or support the effect of probiotics. Interestingly, the use of combination of those components demonstrating a synergistic effect may be even more efficient in the stimulation of intestinal microbiota and protection of animal health. The greatest problem encountered by the scientists who attempt to create synbiotic formulas is selection of appropriate probiotic and prebiotic (high selectivity of action). Feeds containing probiotic organisms are a great hope for that field of the food industry. The hope is even greater considering the fact that consumers do not accept animal products originating from animals in which antibacterial substances had been used. Meeting all expectations requires much work in the field of scientific research, development of innovative technologies and convincing breeders that the spending on prebiotic-containing feed will translate to better production effects and higher quality of animal products, and thus it will guarantee an expected economic profit. It should be underlined that the use of feed additives, such as probiotics, prebiotics and synbiotics is safe, does not have a negative impact on the natural environment, and reduces the demand for antibiotic-based growth stimulators. However, the mechanisms of action of probiotic organisms, prebiotics, as well as their combinations in synbiotics, require further studies.
